# A Graphene-Coated Mo Tip Array for Highly-Efficient Nanostructured Electron Field Emitters

**DOI:** 10.3390/mi9010012

**Published:** 2017-12-29

**Authors:** Ningli Zhu, Jing Chen, Hai Deng, Yunsong Di

**Affiliations:** 1 Aisino Corporation Inc., Beijing 100195, China; denghai@aisino.com; 2 Institute of Microelectronics, Peking University, Beijing 100871, China; j.chen@pku.edu.cn; 3 Department of Engineering, University of Cambridge, Cambridge CB3 0FA, UK; 4 College of Computer Science and Engineering, Nanjing University of Aeronautics and Astronautics, Nanjing 210016, China; 5 Department of Electrical Engineering, Florida International University, Miami, FL 33174, USA; 6 School of Physics Science and Technology, Nanjing Normal University, Nanjing 210046 China; 06171@njnu.edu.cn

**Keywords:** graphene, molybdenum, tip array, field emission

## Abstract

An efficient electron field emitter based on a monolayer graphene coated well aligned Mo tip array has been designed, fabricated, and evaluated. The advantages of this hybrid nanostructure film morphology are explored and discussed. Efficient and stable field emissions with low turn-on fields have been observed with the new devices. It is further found that the combination of graphene and Mo tip array leads to significant improvements in efficiency for the nanoscale heterostructure emitters.

## 1. Introduction

Electron field emission is a quantum tunneling phenomenon whereby electrons are emitted from a solid surface which is affected by a strong electric field [1]. Due to its fast turn-on process, low temperature, ultrahigh brightness, and miniaturized device size, field emission sources are essential elements for a variety of applications such as electron microscopes, flat panel displays, high energy accelerators, X-ray sources, and microwave amplifiers [2,3,4,5,6]. In this paper, the new generation of the field emission devices using graphene-coated Mo tip array is designed, fabricated, and demonstrated.

The materials used for cold cathode emitter must exhibit field enhancing effects internally or externally [7]. Nanostructured materials such as carbon nanotubes [8,9], carbon nanosheets [10,11], graphene [12,13], graphene oxide (GO) [14,15], and sharp nanotips [16,17], are capable to function as more efficient field emitters than traditional emission materials. The graphene and its derivatives are particularly promising due to their unique geometry and electrical properties [15]. It has been reported that the field emission performance of nascent graphene is moderate compared with other nanostructure, such as carbon nanotubes [18]. Conventional method for graphene deposition such as chemical vapor deposition usually lead to graphene flakes lie down or protrude at low angles from the substrate, thereby, limiting their geometrical field enhancement. Planar surfaces with low enhancement factors, contingent on the emitter’s material properties, may need a high turn-on fields of up to 1000 V/μm [19]. High electric fields are undesirable as they can result in deleterious electrical discharge and vacuum breakdown [20].

Significant enhancement of electron field emission capability can only be achievable from the use of sharp edged graphene when the electric field is applied along the sheet due to the local field enhancement at the edge [21]. Practically, field emitters are engineered to have needlelike shapes with sharp tips for dramatic reduction of the turn-on electric fields by several orders of magnitude due to strong local field enhancement at the tips [22]. Thus, tremendous research efforts have been devoted to generate vertically aligned graphene through fabrication technology such as control the CVD growth process [23,24] as to obtain large quantities of free-standing exfoliated graphene from graphite or depositing graphene films with solution processing technology like filtration and spin coating [25,26]. 

In this paper, through device parameter optimizations, we investigated the field electron emission characteristics of graphene-coated Mo tip array that can be used to fabricate an emerging class of highly efficient nanostructured field emitters. Compared with pristine Mo tip array, and graphene-coated flat Mo chip, the proposed graphene-coated Mo tip array was evidently observed with improved emission efficiency and a reduced turn-on field. It is believed that the improved performance is due to the reduction of effective field emission tunneling barrier, which is generated by graphene-metal charge transfer interactions. 

## 2. Design and Theoretical Analysis

Based on Fowler-Nordheim (F-N) theory, electron emission from electron dense surfaces under intense electric fields has been used to investigate the electron-emission behavior of various materials [27,28,29]. Though the degree of its validity at nanoscale remains unclear, it does, nonetheless, coarsely approximate the field emission current (*I*) as a function of the applied electric field (*E*), which can be expressed as [30]:

(1) $$I=\frac{\left(aA\beta^{2}E^{2}\right)}{\varphi }\exp(-b\varphi^{3/2}/\beta E)$$

where $a=1.54\times 10^{-6}$, $b=6.83\times 10^{7}$, A is the emission area, β is the field enhancement factor, φ is the working function and *E* (= *V*/*d*) is the applied electric field. *d* is the cathode to anode distance.

The emission current is highly dependent on both the geometry of the cathode and the material properties. Based on this, at specific fields, lowering the work function of the materials with high aspect ratio emitters is capable of producing higher emission currents [31,32].

**Figure 1:**
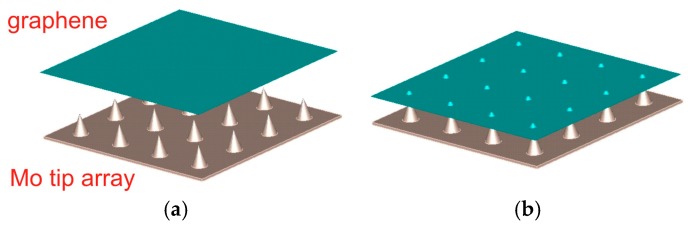
Schematic of the graphene-coated Mo tip array (**a**) before and (**b**) after transfer of the graphene-coated layer.

Among the most promising fabrication technology to increase the field emission performance is the hierarchical development of the cathode using nanoscale surface engineering technology. The emitting surface, comprising primarily of microstructures, is complemented with 1D or 2D nanostructured adlayers [33], which augment the emission efficiency. Molybdenum, along with other refractory metals, is one of the most commonly used field emission materials to date; while graphene and its derivatives are expected to come to the fore due to their unique geometry and electrical properties [34]. The high aspect ratio of monolayer graphene could potentially give dramatic field enhancement, if suitably aligned, and combined with its novel transport properties. In this study, we optimally transfer graphene to Mo tip array for maximum emission efficiency. The design creates an effective way of increasing electric current. Figure 1 shows the process of transferring grapheme into Mo tip array.

There are four dominant factors influencing the level of field emission in the optimization; they are the emitter tip radius *r*, emitter height *h*, inter-emitter pitch *D*, and the global electric field *E*. The parameters *r*, *h*, and *D* are critical for influencing the field enhancement factor *β*. The surface electric field E_s_, which dictates the emission current density *J*, is determined by *E* and *β*.

**Figure 2:**
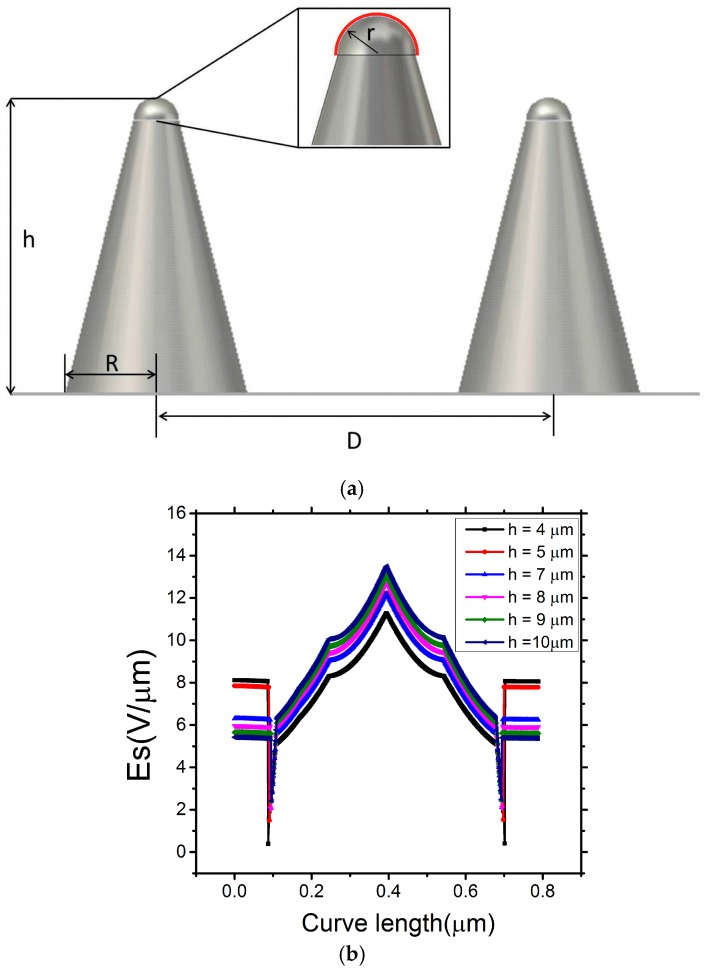
(**a**) Schematic of Mo tip geometry; and (**b**) Electric field verses different height of Mo tip.

The Mo tip surface is designed and fabricated to be, the first order, hemispherical. The bottom radius *R* is set to be 5 µm. The height of the tip is *h*. The distance d between the anode and the top of the Mo tip is 100 µm. The red line on the surface of the hemisphere, in Figure 2a, denotes the probed surface under study. Figure 2b shows the electric field distribution along the red line as a function of height of the Mo tip, where *r* is 0.25 µm, and the curve length is 0.785 µm. The cathode and anode bias are 0 V and 1000 V, respectively. The maximum surface electric field on the Mo tip, as a function of height, was observed to increase from 11.3 V/µm to 13.6 V/µm given the notable geometry mediated field enhancement. 

**Figure 3:**
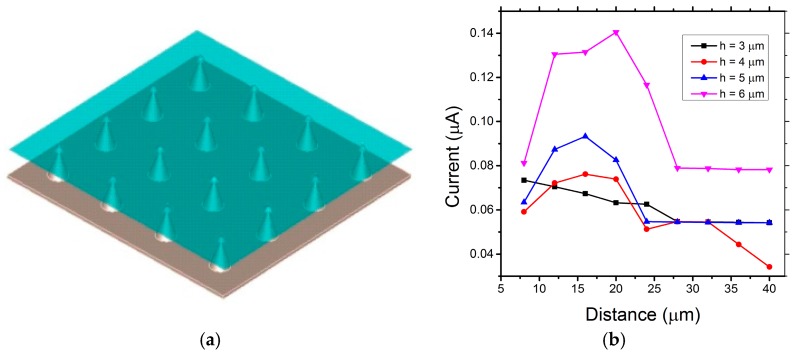
(**a**) Graphene-coated Mo tip array; and (**b**) field emission current as a function of *H* and *D*.

Based on the single Mo tip simulation, the properties of the array of the Mo cold cathode have been investigated herein. Figure 3a shows one possible model of the graphene-coated Mo tip array. A square 4 × 4 array is set on the lower plate (cathode). Graphene are conformably grown exclusively on the upper-most surfaces of the Mo tips. The distance between two individual Mo tip is *D*. All other parameters remain the same as per the earlier single Mo tip model. 

**Figure 4:**
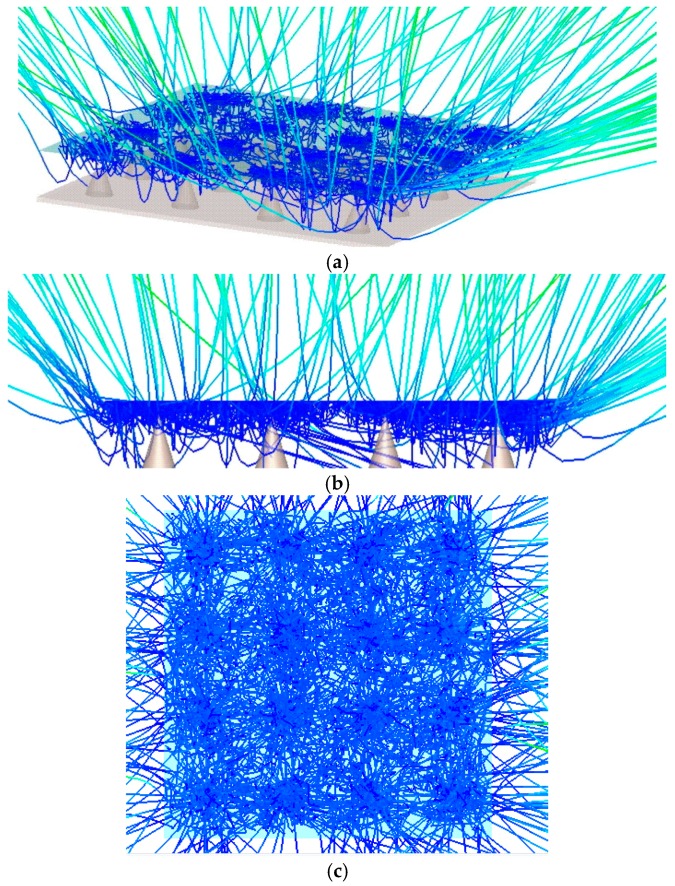
Simulated beam trajectories from graphene-coated Mo tip array (**a**) 3D side view; (**b**) front view; and (**c**) top view of the field emission.

Figure 3b shows the field emission current as a function of *h* and *D* for a radius of 0.25 µm. The distance between two Mo tips increase from 8 to 40 µm with the tip height increasing from 3 to 6 µm. From the simulation results in Figure 3b, the emission current increases with the incensement of *h*. The emission current reaches the maximum when the distance *D* is approximately three times the height *h*. Compared with our experimental results below, upon geometrical optimization, the current was observed to increase by two orders of magnitude. Figure 4a–c is the simulated beam trajectories from graphene-coated Mo tip array, with 3D side view, front view and top view of the field emission.

## 3. Fabrication Processes and Morphology Properties

**Figure 5:**
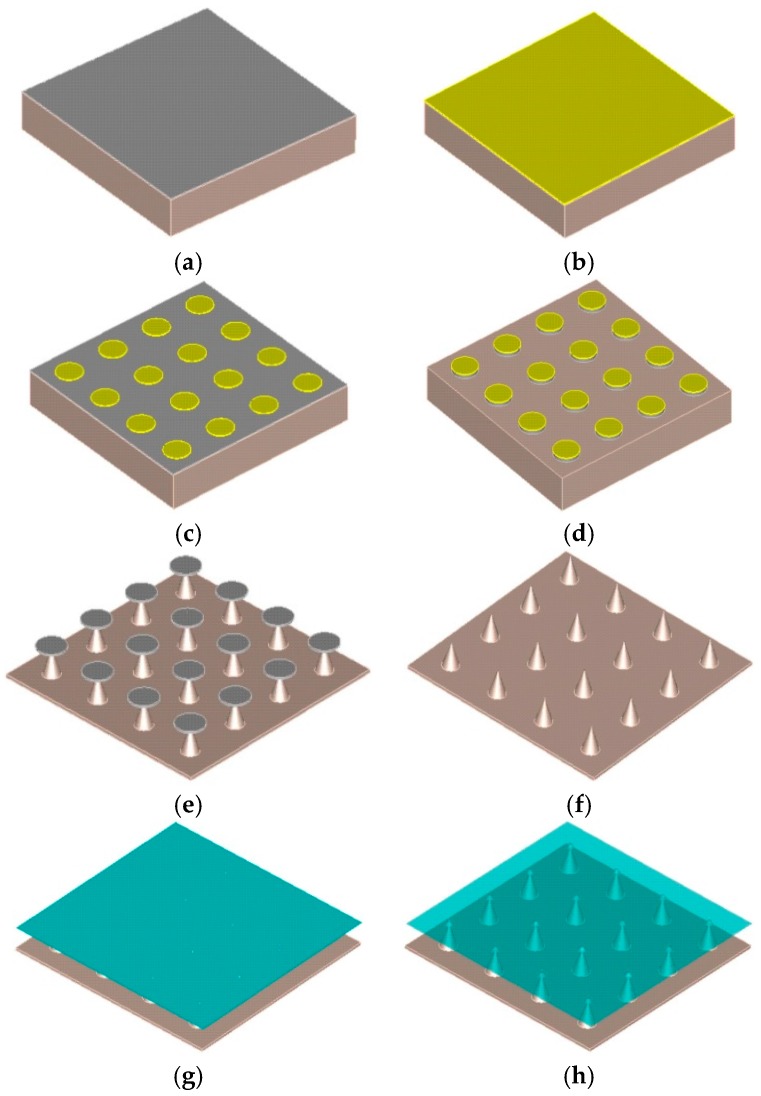
Schematic of the processes flow of transferring the CVD graphene onto the Mo tip array: (**a**) deposition of 300 nm Al; (**b**,**c**) photoresist patterning by optical lithography; (**d**) ICP etching of Al; (**e**) Mo tip forming ICP etch, (**f**) removal of Al mask; (**g**) PMMA/graphene film transfer; and (**h**) PMMA removal by heating.

A schematic description of the fabrication processes flow for the graphene-coated Mo tip array is illustrated in Figure 5 [35]. For brevity, as shown in Figure 5a, a double-side-polished, 400 mm thick, 4-inch high purity (99.95%) Mo wafer was first coated with an evaporated 500 nm thick Al film to form etch mask. The tip array was subsequently patterned by photolithography (Figure 5b,c), with the Al patterned by dry etching in CH_3_F plasma (Figure 5d) [36]. The Mo tip array was etched using an anisotropic SF_6_ dry etch in a commercial ICP etcher (Sentech PTSA 500, SENTECH Instruments GmbH, Berlin, Germany) (Figure 5e). Residual Al was removed by ultrasonication (15 W) for 60 s (Figure 5f). PMMA/graphene films were transferred onto the Mo tips (Figure 5g) by standard PMMA transfer [37], outlined later, with the PMMA subsequently removed by heating to obtain the sample with graphene uniformly covering the Mo tips (Figure 5h).

**Figure 6:**
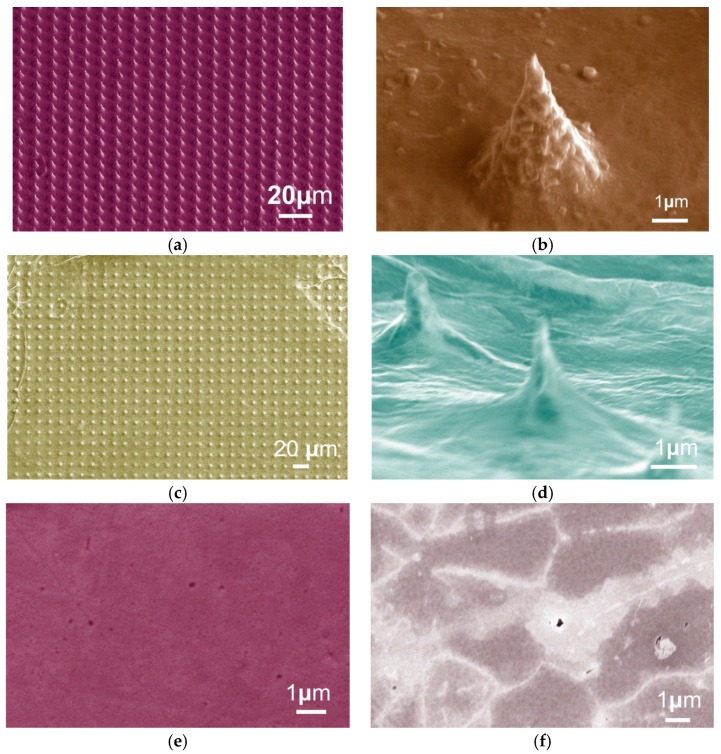
SEM graph of (**a**) Mo tip array; (**b**) perspective view of single Mo tip; (**c**) Mo tip array with grapheme; (**d**) perspective view; (**e**) bulk Mo; and (**f**) bulk Mo with graphene.

A Mo tip array, of 10 μm pitch with *D*/*h* ≈ 1.6, was implemented with good uniformity and a high aspect ratio ≈ 1.8, shown in Figure 6a. The side view of single Mo tip with a tip radius of about 91 nm, as shown in Figure 6b [35]. The graphene functionalized Mo tips were shown in Figure 6c,d. Figure 6d shows the side view of new cones. Upon coating Mo tip with the CVD graphene, we note the occurrence of new conical surface features, where the dimensions of these new cones are comparable to those of the original Mo tips, though the height of the new cones are somewhat shorter, with *D* remaining broadly the same, thus enlarge the *D*/*h_new_*. From the simulation above, with the enlarged *D*/*h_new_*, the emission current is improved significantly. The planar bulk Mo chip before and after being functionalized with graphene was characterized by scanning electron microscopy, as shown in Figure 6e,f. 

**Figure 7:**
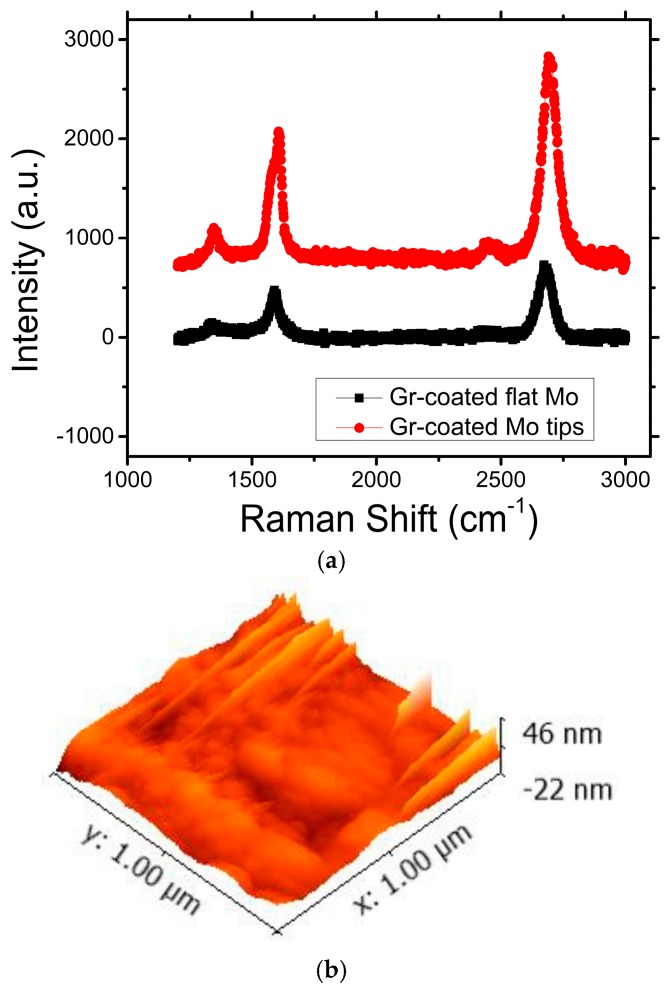
Experimental results of (**a**) the Raman shift of graphene-coated flat Mo chip and graphene-coated Mo tips; and (**b**) surface roughness of the graphene on the Mo tips.

Raman shifts of the graphene-coated Mo tips and graphene-coated flat Mo chip are shown in Figure 7a [35]. The increase in the *I_D_*/*I_G_* ratio of the graphene-coated Mo tips and SEM micrographs indicates an enhancement in defect density in the graphene-coated Mo tips, likely induced by the transfer process and high basal plane stresses induced at the Mo tip apexes. Such defects likely provide additional new emission sites as the variation in field is much larger at defects, edges, and ripples at the atomic scale, which leads to larger local electric fields. The surface roughness of the graphene sheets on the Mo tips, as shown in Figure 7b, predicts the wrinkles on the graphene surface.

## 4. Experimental Results and Discussions

The samples were measured at a base pressure of <10^−7^ mbar in a custom-built, automated field emission system. All measurements were performed at a cathode-to-anode distance of 1 mm. An ITO/glass anode was positioned adjacent to the emitting surface and the emission area of all the three samples was 12.5 mm^2^. The emission characteristics were measured from 0–5 kV, at 50 V increments, with spectra consisting of both up and down sweeps. The emission current was averaged (*n* = 3) at each bias. 

**Figure 8:**
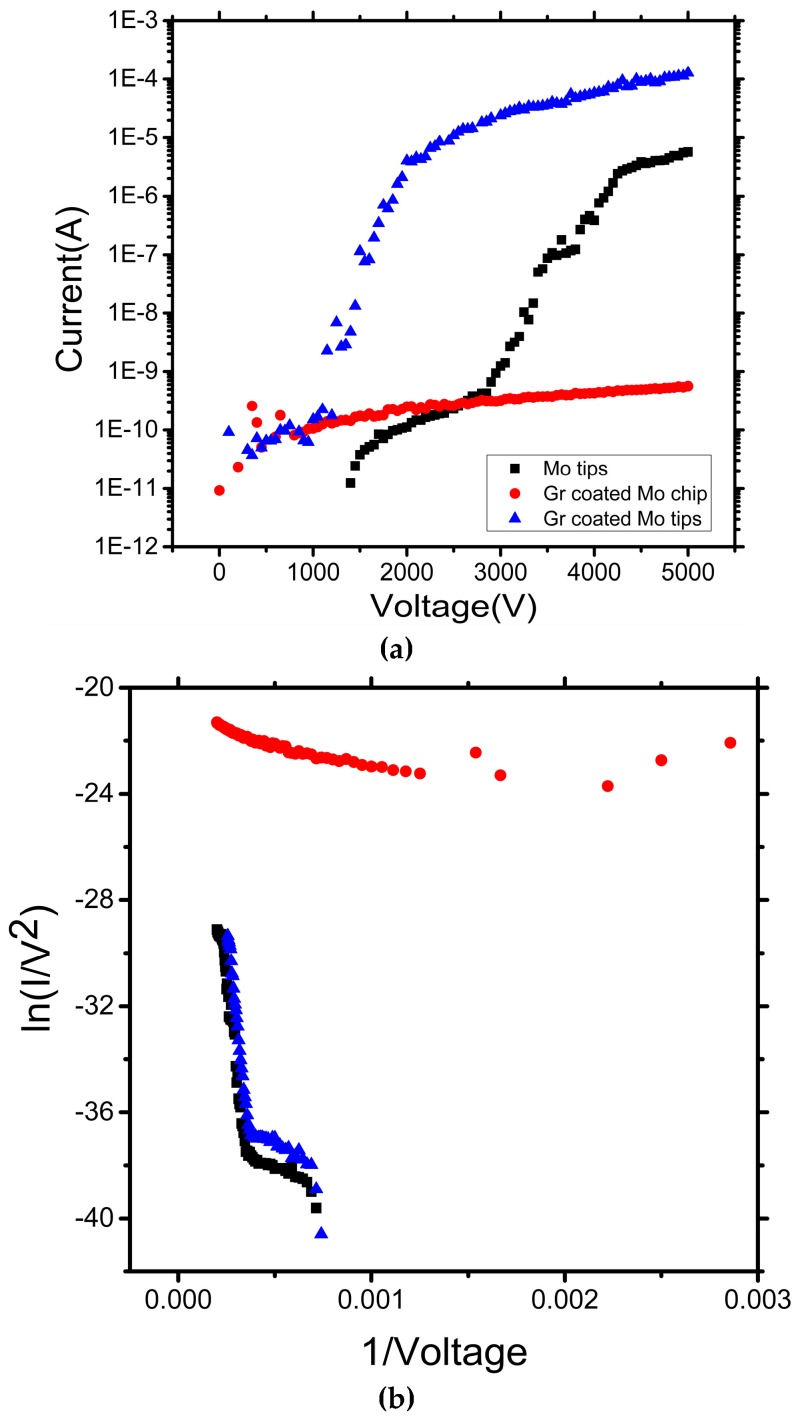
(**a**,**b**) I-V curves of Mo tips, graphene-coated Mo chip and graphene-coated Mo tip array cathodes. (**b**) Corresponding FN plots.

Three different FE cathodes were measured and compared: pristine Mo tip array, graphene-functionalized Mo tip array, and graphene-coated flat Mo chip. Typical I-V characteristics are shown in Figure 8a, with the corresponding F–N plots shown in Figure 8b [35]. 

The maximum emission current of graphene-coated Mo tip array was 1.27 × 10^−4^ A, around an order of magnitude greater than the maximum emission current of the unfunctionalized Mo tips (5.70 × 10^−6^ A). There was little measurable emission current above the SMU noise floor from the graphene sheets on flat Mo chip, even under the maximum applied electric field of 5 × 10^6^ V/m, as shown in Figure 8a. We observed that the maximum emission current of the fabricated devices was remarkedly enhanced when the graphene-coated Mo tips field emitters are used as the cathodes. The observed enhancement of the FE performance may be attributed to the gradual formation of the increased electric field at the wrinkled graphene protrusions [38]. 

Controlling the tip areal density was also important in determining the field emission performance, as electron screening from neighboring tips must be minimized. Our simulations suggest that the emission current density reaches a maximum when the distance *D* is approximately three times the height h. For the Mo tip array, *D*/*h* ≈ 1.6, suggesting that the cones are overly closely packed for the optimal emission. Upon coating Mo tip with the CVD graphene, we noted the occurrence of new conical surface features, where the dimensions of these new cones are comparable to those of the original Mo tips, though the height of the new cones are somewhat shorter, with *D* remaining broadly the same, thus enlarging *D*/*h_new_* and, thus, adjusting the emission current density.

Additionally, after coating Mo tip array with graphene, field emission occurred at considerably lower turn-on fields. We attribute this to the possible formation of a triple junction between Mo, graphene, and vacuum [39] coupled to the increased areal density of atomically sharp, though admittedly small, protrusions. In the triple junction, the surface potential undergoes a step change at the junction between the graphene and Mo due to the difference in work function. This surface potential irregularity may modify the local potential in the vicinity of the junction. In this regard, it is possible to explain the electron emission for the graphene functionalized Mo tips as follows: The emission from the Mo tips/graphene/vacuum triple junction occurs due to an enhancement of the applied field brought about by an augmented aspect ratio, which is possibly further amplified by triple junction affects. The graphene-coated planar Mo chip aspect was especially low, and triple junction effects may, indeed, enhance the observed emission. It appears that aspect ratio effects dominate the emission improvements greatly. 

## 5. Conclusions

In this paper, an innovative design for using a hybrid graphene-coated Mo tip array for efficient field emission is demonstrated, designed and fabricated. Both simulation and testing results demonstrate the new present methodology leads to lowering of the turn on field and enhanced maximum emission currents. The maximum emission current of the graphene-functionalized Mo tip array is 22 times larger than the pristine Mo tip array. The feasibility of depositing wrinkled graphene sheets in large scale will allow further investigation of the new devices, as well as exploiting their unique 2D structure for many potential applications. This work could pave the way for the design and applications of future electron emission 2D heterostructure nano devices.
